# Gly Betaine Surpasses Melatonin to Improve Salt Tolerance in *Dalbergia odorifera*

**DOI:** 10.3389/fpls.2021.588847

**Published:** 2021-02-09

**Authors:** El Hadji Malick Cisse, Ling-Feng Miao, Fan Yang, Jin-Fu Huang, Da-Dong Li, Juan Zhang

**Affiliations:** School of Ecological and Environmental Sciences, Center for Eco-Environmental Restoration Engineering of Hainan Province, Key Laboratory of Agro-Forestry Environmental Processes and Ecological Regulation of Hainan Province, Hainan University, Haikou, China

**Keywords:** alternative oxidase, glycine betaine, melatonin, redox homeostasis, salt tolerance

## Abstract

Salinity is one of the most serious factors limiting plant growth which can provoke significant losses in agricultural crop production, particularly in arid and semi-arid areas. This study aimed to investigate whether melatonin (MT; 0.05 and 0.1 mM), which has pleiotropic roles, has a better effect than glycine betaine (GB; 10 and 50 mM) on providing salt tolerance in a woody plant *Dalbergia odorifera* T. Chen. Also, the alternative oxidase activity (AOX) in plant subjected to MT or GB under salinity (150 and 250 mM) was evaluated given that the effect of exogenous MT or GB on AOX has not been reported yet. The results showed that the exogenous application of GB on the seedlings of *D. odorifera* increased the plant growth parameters, relative water content, total of chlorophyll content, and carotenoid content compared with well-watered and MT treatments. Under severe salinity, the seedlings subjected to GB showed, a significant enhancement in water use efficiency, transpiration, and net photosynthetic rate regardless to MT-treated seedlings. The levels of proline and soluble sugar in the seedlings treated with MT or GB decreased significantly under mild and severe salinity correlated with those in salt-stressed seedlings. Furthermore, GB-treated plants exhibited a significant inhibition of malondialdehyde content compared with MT-treated plants. The concentration of thiols and phenolic compounds were significantly enhanced in the leaves of seedlings treated with MT compared with those treated with GB. Under salt stress condition, GB scavenged significantly higher levels of hydrogen peroxide than MT; while under severe salinity, plants subjected to MT showed better scavenging ability for hydroxyl radicals compared with GB-treated seedlings. The results demonstrated also an enhancement of the levels of superoxide dismutase (SOD), guaiacol peroxidase, and AOX activities in seedlings treated with GB or MT compared with salt-stressed plants. The catalase activity (CAT) was increased by 0.05 mM MT and 0.1 mM GB under mild salinity. Meanwhile, the AOX activity under severe salinity was enhanced only by GB 50 mM. The findings of this study suggested that GB-treated seedlings possessed a better salt tolerance in comparison with MT-treated seedlings.

## Highlights

- GB and MT protect the homeostasis by decreasing the ROS, EL, and MDA.
- GB and MT promote the antioxidant activities including AOX.
- GB improves better salt tolerance compared to MT.

## Introduction

Salinity is one of the most threatening abiotic stresses that can cause significant losses in agricultural crop production. Salinity can trigger an oxidative stress on plants at the sub-cellular level by overproduction and accumulation of reactive oxygen species (ROS), such as hydrogen peroxide (H_2_O_2_) and hydroxyl free radical (⋅OH) (Acosta-Motos et al., 2017; Hernández, 2019). Salt stress deprives plants to access soil water by increasing the osmotic strength of the soil solution. It can severely affect plant growth and photosynthetic apparatus by promoting ion toxicity and oxidative stress (Volkmar et al., 1998). There have been numerous studies to investigate the effect of exogenous application of melatonin (MT) or glycine betaine (GB) in plants under salt stress. Results showed that both of them can improve salt tolerance in various plant species. The glycine betaine, also named osmoprotectant (compatible solute) is belongs to the group of osmolytes that are present in all living organisms. GB (quaternary ammonium compound) is one the most well-known osmoprotectant and confers tolerance to abiotic stress in different plants (McNeil et al., 1999; Li et al., 2019). During abiotic stresses, GB can protect plant cells from oxidative stress by enhancing the antioxidant system (Demiral and Türkan, 2004; Malekzadeh, 2015). Meanwhile, the melatonin (N-acetyl-5-methoxytryptamine) plays also a vital role in plant stress tolerance. MT is an indolic compound derived from serotonin (5-hydroxytryptamine) and a multi-regulatory molecule that has many specific functions in plant physiology (Arnao and Hernández-Ruiz, 2019). Indeed, MT can improve homeostasis and photosynthesis and regulates gene expression in response to salt stress in plants (Szafrańska et al., 2016; Martinez et al., 2018). MT can activate the antioxidant systems in response to abiotic stresses, including salt stress. This phenomenon has been demonstrated by different authors in different species, such as soybean, rice, maize, radish, cucumber, papaya, and watermelon (Li et al., 2019). In rapeseed and cucumber, MT can significantly decrease the concentration of ROS induced by abiotic stress (Li et al., 2018). Furthermore, the literature review shows that the effect of salinity on woody plants is omnipresent and becomes disturbing with global climate change. Plants naturally establish diverse mechanisms to survive under certain salt concentrations in soil, which can be resulting in their death or growth inhibition (Flowers and Yeo, 1989).

*Dalbergia odorifera* T. Chen, a woody plant also named as fragrant rosewood, is a semi-deciduous perennial tree widely distributed in tropical areas, particularly in China. This plant is endemic to Hainan Island and belongs to the family of Leguminosae; this plant is threatened by habitat loss and overexploitation due to timber usage (Liu et al., 2019).

The demand for high-quality seedlings of fragrant rosewood in southern areas increases for use in forest establishment (Li et al., 2018). Many studies have investigated the positive effects of MT and GB on crop plants subjected to diverse abiotic stresses, but few works were conducted on woody plants. Thus, the overall goal of this present work was to investigate how *D. odorifera* seedlings respond to exogenous GB or MT. Moreover, the fact that MT might be considered as a phytohormone because of its structure and functions similar to auxin (IAA), it can improve the redox homeostasis and has a strong power to regulate plant growth (Arnao and Hernández-Ruiz, 2019). Meanwhile, GB promotes growth and survival of plants counteracting metabolic dysfunctions caused by stress (Annunziata et al., 2019). Thus, it is of interest to know which molecule is more effective to provide salt stress tolerance in plant. Hence, we hypothesized that MT might improve salt tolerance better than GB. Many studies have revealed that the alternative oxidase (AOX) gene expression is enhanced by different environmental stresses, including severe salinity. Furthermore, AOX is an antioxidant enzyme that has the same role as guaiacol peroxidase (POD) or superoxide dismutase (SOD) in regulating the synthesis of ROS, such as superoxide and H_2_O_2_. Considering that exogenous GB and MT increases the antioxidant enzyme activities in diverse species under abiotic stresses. In this study, we also hypothesized that the AOX activity would be enhanced by exogenous GB and MT application under salt stress.

## Materials and Methods

### Plant Materials and Experimental Design

The layout of the trial was a factorial experiment in a completely randomized design using two factors and six replicates. The factors included (i) salinity level (Sodium chloride) (11.0 and 18.3 dS/m), (ii) MT and GB level (MT0.05 and 0.1 mM; GB 10 and 50 mM). The study was carried out in a greenhouse at Hainan University (20° 03′ 22.80″ N, 110° 19′ 10.20″ E) during April–June 2019. Six-month-old native seedlings of *D. odorifera* were collected in Ledong County (18° 44′ 52″ N, 109° 17′ 31″ E), Hainan province. The seedlings were transplanted in pots and grown under natural light conditions. The pots (10 cm in height and 12 cm in diameter) were filled with red soil mixed with 30% of sand. After a month of growth, healthy seedlings with approximately the same height and size of twig were selected. Treatments were designed as follows: (1) **CK:** control, well-watered conditions; (2) **M1**: 0.05 mM MT; (3) **M1S1**: 0.05 mM MT and 150 mM salt solution; (4) **M1S2**: 0.05 mM MT and 250 mM salt solution; (5) **M2**: 0.1 mM MT; (6) **M2S1**: 0.1 mM MT and 150 mM salt solution; (7) **M2S2**: 0.1 mM MT and 250 mM salt solution; (8) **G1**: 10 mM GB; (9) **G1S1**: 10 mM GB and 150 mM salt solution; (10) **G1S2**: 10 mM GB and 250 mM salt solution; (11) **G2**: 50 mM GB; (12) **G2S1**: 50 mM GB and 150 mM salt solution; (13) **G2S2**: 50 mM GB and 250 mM salt solution (14) **S1**: 150 mM salt solution; (15) **S2**: 250 mM salt solution. The treatments were selected on the basis of the articles published by Ye et al. (2016), Kolář et al. (2003), and Wei et al. (2015). GB and MT solutions were applied first on the substrate (soil) and then sprayed on the leaves 5 days before the salinity treatment and every 2 days during the salt treatment (once in the morning). The seedlings were watered with clean water each day during the experiment to cope with high temperature and light condition in the greenhouse. The seedlings under well-watered and salt stress without MT or GB were sprayed with distilled water. After 25 days of treatment, leaves were harvested and cleaned with distilled water for physiological and biochemical analyses.

### Growth Measurements

The leaf length and area were measured by a portable area meter LI-3000C (Li-COR, United States). Increments in leaf number, plant height, and stem diameter were recorded at the last treatment day. Data were collected from six seedlings in each treatment, and leaves were harvested and kept at −80°C. Relative water content, dew point water potential, and electrolyte leakage were measured on the harvest day.

### Photosynthetic Parameter Measurement

Pigments content was determined in leaves by colorimetric method at absorbance of 663,646, and 470 nm with 80% acetone. Approximately 0.2 g leaf tissue was ground in 10 mL of 80% acetone (v/v), then the extract was centrifuged at 4,000 × *g* at 4°C for 10 min, and the supernatant was used for spectrophotometer readings. The concentration of pigments in each sample was calculated according to the following equations (Lichtenthaler and Wellburn, 1983):

Chlorophyll⁢a=12.21⁢A⁢663-2.81⁢A⁢646

Chlorophyllb=20.13A646-5.03A663

Carotenoid=(1000⁢A⁢470-3.27⁢Chlorophyll⁢a⁢-104⁢Chlorophyll⁢b)/229

Net photosynthetic rate (Pn), intercellular carbon dioxide (Ci), stomatal conductance (Gs), transpiration rate (Trr), and water use efficiency (Wue) were measured with a TP-3051D photosynthetic apparatus (Zhejiang Top Instrument Co., Ltd.). Measurements were conducted on six seedlings (three leaves each seedling) in each treatment at 10:00–12:00 am according to Yang et al. (2010).

### Determination of Osmolytes, Thiols, Phenols, and Proteins

**Proline** quantification was conducted by colorimetric method described by Bates et al. (1973) with some modifications. Sample of 500 mg leaves was grounded into liquid nitrogen and homogenized in 10 mL of 3% aqueous sulfosalicylic acid. About 2 mL of the filtered homogenate was mixed with acid-acetic ninhydrin reagent, and added to 2 mL of glacial acetic acid then incubated at 100°C for 1 h. The reaction was stopped by cooling the samples on ice. The chromophore-containing phase was extracted with 4 mL of toluene and the absorbance was measured at 520 nm. The proline concentration was determined using a standard curve.

**Total soluble sugar content** was determined by anthrone method (0.2% anthrone) according to Yemm and Willis (1954). About 2 mL of the reagent was added to 1 mL of the sample. Absorbance was read at 630 nm and a standard graph was used to calculate the concentration of soluble sugar in each sample.

**Thiols** react with 5, 5′-dithiobis (2-nitrobenzoic acid) DTNB to form 2-nitro-5-thiobenzoic acid (TNB), which turns yellow in alkaline medium and absorbs at 412 nm (Ellman, 1959).

A standard curve was used to determine the concentration of thiols. In brief, 0.5 M aqueous Tris solution (250 mL), DTNB aqueous solution (DTNB 10 mM; EDTA 20 mM for 25 mL), and 3% sulfosalicylic acid solution were prepared, and glutathione reduced aqueous solution 1 mM (50 mL) was prepared for the standard curve. About 1 mL of the standard solution or sample reacted with 50 μL of DTNB and the added with 1 mL of 0.5 M Tris. Absorbance was read at 412 nm after 30 min.

**The total of phenol** content was estimated according to the method of Swain and Hillis (1959), which was based on the Folin–Ciocalteu reagent. The oxidation of phenols reduces this reagent to a mixture of blue tungsten and molybdenum oxides. The intensity of the color is proportional to the rate of oxidized phenolic compounds. Leaves were crushed in 80% ethanol (v/v) and stirred hot (80°C) for 30 min. Ethanol was evaporated, and the residue was dissolved in 20 mL of distilled water. About 1 mL aliquot of the sample was added with 7.5 mL of distilled water and 0.5 mL of Folin’s reagent and stirred vigorously. After 3 min, 1 mL of saturated Na_2_CO_3_ solution (40%) was added to the tube. After 1 h, absorbance was read at 725 nm, and the quantity of phenols was determined according to the following formula: A725 × V/Fw (V, volume; Fw, fresh weight).

**Soluble protein** content was determined by Bradford method; the reagent was prepared with 100 mg of Coomassie Brilliant Blue G-250 diluted in 50 mL of ethanol and 100 mL of 85% phosphoric acid was added. The final solution was completed with distilled water up to 1,000 mL and the reagent was filtered through Whatman filter paper. About 100 mg leaf samples were homogenized in 2 mL of phosphate buffer (pH 7.8). An aliquot of volume *v* (μL) was mixed with 1 mL of the reagent and absorbance was read at 595 nm.

### Measurement of Malondialdehyde and ROS Accumulation

**Malondialdehyde (MDA)** was quantified by colorimetric method using 200 μL of samples with 800 μL of 20% (w/v) TCA containing 0.5% (w/v) 2-thiobarbituric acid in accordance with the method of Yang et al. (2010) with some modifications. About 200 mg of sample was homogenized in 5.0 mL of 5% (w/v) TCA and centrifuged at 12,000 × *g* for 10 min. About 4 mL of 20% trichloroacetic acid (TCA), containing 0.5% thiobarbituric acid (TBA), was mixed with 1 mL of the supernatant, incubated at 95°C for 30 min, cooled on ice, centrifuged at 8,000 × *g* for 15 min, and absorbance was read at 532 nm. Then absorbance was read at 532 nm and corrected by subtracting the value obtained at 600 nm (non-specific absorbance).

**H_2_O_2_** was quantified according to the method of Yang et al. (2010). Approximately 200 mg of fresh leaves were grounded and homogenized in 0.1% trichloroacetic acid then centrifuged at 6,000 × *g* for 15 min at 4°C. A volume of supernatant was incubated in the presence of potassium iodide added to 10 mM buffer solution. Absorbance was read at 390 nm, and the concentration of H_2_O_2_was estimated via a standard curve.

**The concentration of ⋅OH** was estimated with a colorimetric Hydroxyl Free Radical Scavenging Capacity Assay Kit (Nanjing Jiancheng Bioengineering Institute, China) based on the Fenton reaction, which is the most common chemical reaction that can produce ⋅OH. Approximately 100 mg of grounded leaf was mixed with 2 mL of potassium phosphate buffer (pH 7.8) and centrifuged at 8,000 × *g* for 10 min. Absorbance was recorded at 550 nm, and phosphate buffer solution was used as extraction solution.

### Determination of Antioxidant Enzyme Activities

In brief, 1 g of fresh leaves were washed with distilled water, weighed and triturated in a mortar at 4°C by using 10 mL of 0.1 M phosphate buffer solution (pH 7.0) containing 0.1 mM EDTA, 0.1 mM ascorbate, and 1% polyvinylpolypyrrolidone (PVPP). The extract was centrifuged for 15 min at 12,000 × *g* and 4°C and used to assay the enzymatic activity of POD.

**Peroxidase activity** was measured following the method described by Fielding and Hall (1978) based on monitoring of guaiacol peroxidase scavenging activity by using guaiacol as hydrogen donor. The increase in absorption caused by guaiacol oxidation by H_2_O_2_ was measured at 470 nm. The reaction mixture contained 10 mM H_2_O_2_, 50 mM phosphate buffer, and 9 mM guaiacol in a total final volume of 3 mL with the sample.

**Catalase activity (CAT)** was measured by the procedure described by Aebi (1984). CAT activity was determined by colorimetric assay at 240 nm by measuring the decomposition of H_2_O_2_. The reaction mixture contained 100 mM phosphate buffer (pH 7.0), 30 mM H_2_O_2_, and 100 μL of crude extract in a total volume of 3.0 mL, CAT activity was measured and recorded by a decrease in absorbance until 0.5–3 min. CAT activity was calculated using a standard curve.

**Ascorbate peroxidase (APX)** and **SOD** were determined in accordance with the manufacturer’s instructions and as described Yang et al. (2010, 2015). Fresh samples were weighed, then minced to small pieces in liquid nitrogen and homogenized with PBS (10 mg tissue to 100 μL PBS). After that, the particulates were removed by centrifugation (10,000 × *g* for 30 min at 4°C) and the assay was immediately performed with the aliquot and the rest of the samples were stored at −20°C. The supernatant was used for SOD and APX analysis by APX test kit and SOD assay kit based on hydroxylamine method (Nanjing Jiancheng Bioengineering Institute, China).

**Alternative oxidase activity** was evaluated using ELISA KIT (JL22749 Plant AOX ELISA KIT; 48T/96T). As one of the terminal oxidases of plant mitochondrial electron transport chain, AOX plays an important role in counterattacking salt stress. The reaction uses a purified plant AOX antibody to coat micro strip plate wells to make a solid-phase antibody. The addition of AOX and AOX antibody labeled with HRP to wells gives a complex antibody–antigen–antibody–enzyme complex. After washing completely, the system was added with 3, 3′, 5, 5′-tetramethylbenzidine (TMB) substrate solution. The TMB substrate becomes blue under HRP enzyme-catalyzed reaction. The reaction was terminated by adding sulfuric acid solution, and color change (yellow) was measured spectrophotometrically. For this assay, fresh leaves were collected, weighed, and homogenized with PBS (10 mg of tissues to 100 μL of PBS). The mixture was centrifuged at 1,000 × *g* for 20 min, and the supernatant was carefully collected. The assay was immediately conducted, and absorbance was read at 450 nm. A standard curve was used to estimate the level of AOX.

### Statistical Analyses

The results were expressed as mean ± standard error, and Graph Pad prism 8.0.2 software was used to draw the graphs and analyze the data. All data were subjected to analysis of variance for a factorial experiment in a completely randomized design. Statistically significant differences between means were determined at *p* ≤ 0.05 using Tukey’s HSD (honestly significant difference) test. The principal component analysis was performed by Graph Pad prism 9.0.0. The heat-map was generated by using the 9 PCAs from loadings data.

## Results

### Effect of GB and MT on Seedling Growth Under Salt Stress

Salt stress (150 and 250 mM) significantly decreased the plant height, leaf area, and the number of leaves (*p* ≤ 0.05). The exogenous application of GB and MT enhanced the growth traits of *D. odorifera* under salt stress compared with the salt-stressed seedlings. In general, GB at all levels increased the plant height, leaf area, leaf length, and leaf number compared with the other treatments including the control (Supplementary Figures 1–3 and Table 1). The application of GB and MT under salinity decreased significantly the electrolyte leakage and dew point water potential, and increased strongly the relative water content in leaves. Except for G1S1, GB treatments increased significantly the relative water content even under salt stress. The relative water content in control seedlings was 77.59%, and it were 82.43, 76.67, 78.86, 84.84, 81.42, and 77.67% for G1, G1S1, G1S2, G2, G2S1, and G2S2, respectively (Table 1).

**TABLE 1:** Variations of plant height increment (PHI), number of leave increment (NLI), stem of diameter increment (SDI), relative water content(RWC), dew-point water potential (DWP), electrolyte leakage (EL), and leaf area (LA) in *D. odorifera* seedlings subjected to melatonin and glycine betaine under salinity (150 and 250 mM).

Treatment NaCl (mM)	GB/MT	PHI (cm)	NLI	SDI (mm)	RWC (%)	DPW (Mpa)	EL (%)	LA (cm^2^)
0 mM	−MT/GB(Ck)	48.02.82c	81.82cde	0.30.08bc	77.591.62cd	−3.520.80ab	25.613.83h	18.291.03c
	++M1	44.02.58d	70.81def	0.20.07c	77.652.83cd	−4.030.18abc	26.710.96g	18.401.16c
	+M2	40.01.44e	91.80cd	0.40.08ab	77.652.47cd	−3.952.83abc	28.902.94ef	10.981.08i
	+G1	52.51.21b	122.44ab	0.20.08c	82.431.64ab	−2.700.69a	17.250.80j	15.811.02f
	+G2	51.00.11b	100.83cd	0.40.11ab	84.844.86a	−3.923.81abc	26.711.47g	12.521.01h
150 mM	−MT/GB (S1)	37.01.63f	61.41efg	0.30.01bc	70.903.61e	−7.031.63d	32.214.13c	9.011.44k
	+M1	35.51.29fg	61.41efg	0.30.06bc	72.931.63e	−6.451.82cd	29.596.28de	7.961.11m
	+M2	46.51.19cd	80.81cde	0.40.08ab	70.426.09e	−5.361.24bcd	29.121.18def	22.851.01b
	+G1	52.51.73b	122.44ab	0.30.01bc	76.673.26d	−4.320.95abc	28.340.73f	24.101.01a
	+G2	41.00.21e	131.63a	0.50.02a	81.425.21abc	−3.630.77ab	22.760.97i	16.882.14d
250 mM	−MT/GB (S2)	31.01.36h	40.71g	0.250.01c	71.222.12e	−14.981.63f	36.280.76a	8.301.01l
	+M1	34.00.81g	50.91fg	0.30.14bc	71.711.72e	−11.440.92e	36.024.36a	6.680.16n
	+M2	44.51.29d	40.84g	0.40.08ab	72.812.96e	−5.365.27ab	36.145.24a	12.932.12g
	+G1	59.00.95a	102.16cd	0.40.02ab	78.861.75cd	−4.303.97abc	29.921.81d	16.211.58e
	+G2	58.01.63a	71.82def	0.40.09ab	77.675.95cd	−5.900.77bcd	34.061.62b	9.941.33j

*Values are expressed as mean ± SD. The different letters indicate significant differences between treatments (*p* < 0.05) based on Tukey’s multiple comparison test. The *F*-test between salinity factor and GB/MT: plant height increment *P* < 0.0001, number of leaf increment *P* = 0.0236, stem of diameter increment *P* = 0.0716, water content *P* = 0.0012, dew-point water *P* < 0.0001, electrolyte leakage *P* < 0.0001, and leaf area *P* < 0.0001.*

### Effect of GB and MT on Photosynthetic Parameters

**FIGURE 1:**
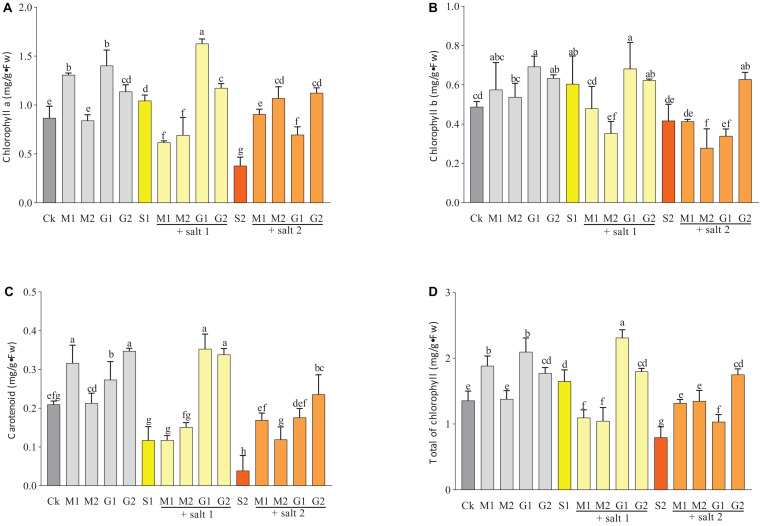
Effects of melatonin and glycine betaine on chlorophyll a **(A)**, chlorophyll b **(B)**, carotenoids **(C)**, and the total of chlorophyll contents **(D)** under salt stress (150 and 250 mM). The bars on the top show SD, and different letters indicate significant differences according to Tukey’s multiple comparison test (*p* < 0.05).

As shown in Figure 1, salinity decreased the contents of chlorophyll a, chlorophyll b, and carotenoids in seedlings treated with salinity. The addition of GB during stress increased the concentration of these pigments. Seedlings exposed to G1 possessed significant increase in the content of chlorophyll a and b compared with control seedlings. The carotenoid content significantly increased when the seedlings were subjected to G2 and G2S1. The seedlings exposed to GB treatment showed a better salt tolerance than those subjected to MT treatments. In severe salt stress (250 mM), the MT treatments increased the total of chlorophyll and carotenoids contents in leaves.

**FIGURE 2:**
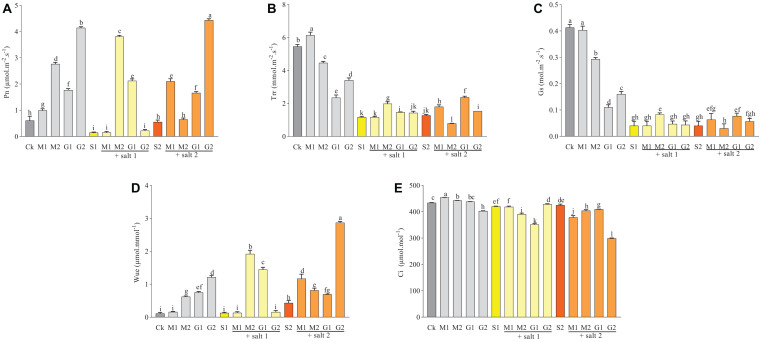
Variations of Pn **(A)**, Trr **(B)**, Gs **(C)**, Wue **(D)**, and Ci **(E)** in *D. odorifera* subjected to melatonin and glycine betaine under salt stress (150 and 250 mM). The bars on the top show SD, and different letters indicate significant differences according to Tukey’s multiple comparison test (*p* < 0.05).

Figure 2 showed that the Pn, Trr, Gs, and Wue in *D. odorifera* seedlings treated with GB or MT significantly increased under severe salt stress. Meanwhile, the Trr in seedlings treated with 0.1 mM MT decreased significantly compared with seedlings treated with S2. The Ci in leaves was reduced significantly when the seedlings were subjected to MT and GB under severe salinity. In mild salt stress (150 mM), the Trr, Pn, and Wue increased in seedlings treated with MT and GB compared with those in seedling treated with S1. Furthermore, treatment with 0.1 mM MT increased the Gs compared with S1. Seedlings that were not subjected to salt stress and treated with MT and GB showed an increase in the Wue and Pn. Based on the matrix table of Tukey’s HSD, seedlings treated with GB showed a significant increase in the Wue, Trr, and Pn compared with seedlings treated with MT under severe salt stress. However, plants subjected to 0.1 mM MT exhibited increase in the Pn, Trr, Gs, and Wue compared with the seedlings treated with GB under mild salt stress (Supplementary Table 1).

### Accumulation of Osmolytes, Thiols, Phenols, and Proteins

**FIGURE 3:**
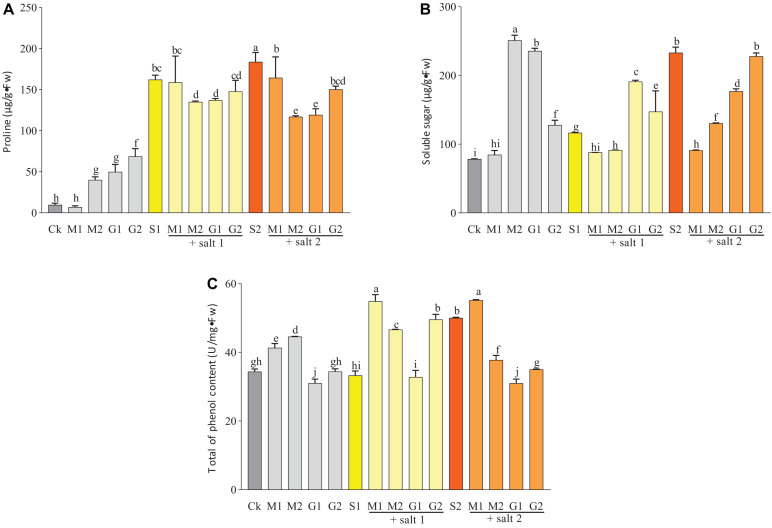
Total of phenol **(A)**, proline **(B)**, and soluble sugar **(C)** contents in *D. odorifera* seedlings submitted to MT and GB under salinity (150 and 250 mM). The bars on the top show SD, and different letters indicate significant differences according to Tukey’s multiple comparison test (*p* < 0.05).

Under well-watered conditions, the total of phenol content was increased by MT but decreased by treatment with 10 mM GB. Under mild and severe salt stress, only MT-treated seedlings showed a significant increase in the total of phenol content (Figure 3).

**FIGURE 4:**
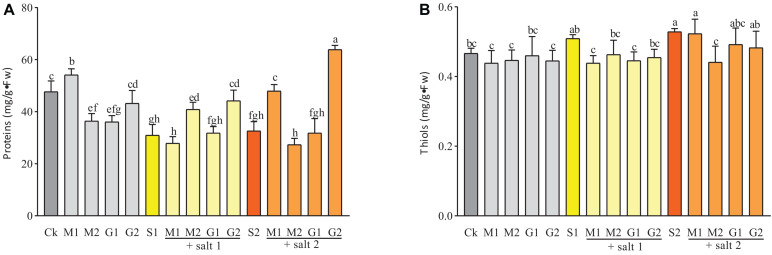
Proteins **(A)** and thiols **(B)** contents in *D. odorifera* seedlings submitted to MT and GB under salinity (150 and 250 mM). The bars on the top show SD, and different letters indicate significant differences according to Tukey’s multiple comparison test (*p* < 0.05).

The concentration of thiols was decreased slightly by MT and GB treatments under salinity or without stress compared with the control. Under mild salinity, seedlings treated with MT or GB showed a similar concentration of thiols. However, under severe salt stress or well-watered condition, the concentration of thiols was significantly enhanced in the leaves of seedlings treated with MT compared with GB-treated seedlings (Figure 4).

As shown in Figure 3, the proline and soluble sugar contents in the leaves of *D. odorifera* seedlings treated with MT (M1S1, M2S1, M1S2, and M2S2) and GB (G1S1, G2S1, G1S2, and G2S2) decreased significantly under mild and severe salinity compared with those in seedlings that were only treated with salt solution. Meanwhile, the soluble sugar content in seedlings subjected to GB under mild salinity was significantly increased compared with that in seedlings treated with 150 mM salt solution. When the seedlings were treated with MT or GB under well-watered condition, the proline and soluble sugar contents in the leaves increased strongly compared with those in the control group, except for the leaves of seedlings exposed to 0.05 mM MT. At all levels of salinity and well-watered conditions, the MT treatments increased significantly the content of phenols compared with the GB treatments. A comparison with the control of severe salinity group showed that the plants treated with MT had higher concentration of phenols and their concentrations were significantly increased under well-watered conditions. In severe salinity, treatments with 0.05 mM MT and 50 mM GB significantly increased the protein content in seedlings of *D. odorifera* compared with the control group that was subjected to only 250 mM of salt solution. Under mild salinity, the protein content was strongly increased in seedlings treated with 0.05 mM MT or 50 mM GB. Under well-watered conditions, only the plants subjected to 0.05 mM MT showed significant increase in the protein content (Figure 4).

### Reactive Oxygen Species and MDA Accumulation

**FIGURE 5:**
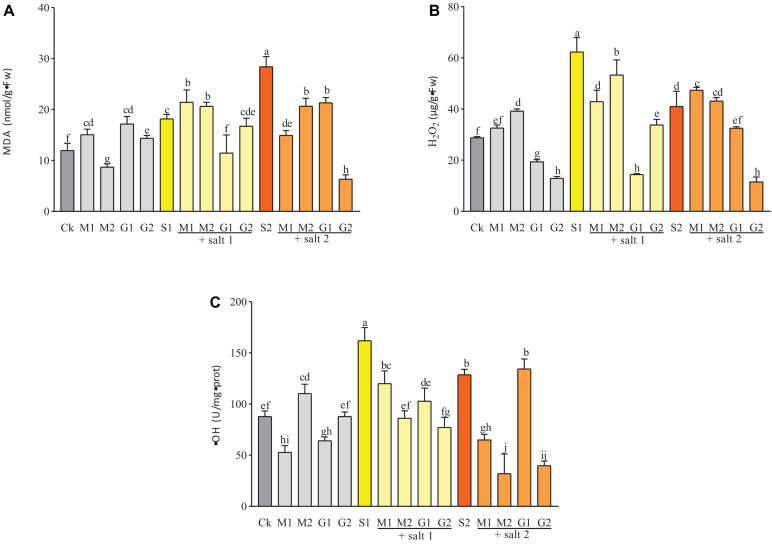
⋅OH **(A)**, H_2_O_2_ **(B)**, and MDA **(C)** contents in *D. odorifera* seedlings submitted to MT and GB under salinity (150 and 250 mM). The bars on the top show SD, and different letters indicate significant differences according to Tukey’s multiple comparison test (*p* < 0.05).

Melatonin and GB decreased the ⋅OH content under salt stress, except in 10 mM GB-treated seedlings under severe salinity (Figure 5). GB significantly decreased the ⋅OH and H_2_O_2_ contents. Meanwhile the ⋅OH concentration in plants treated with 50 mM GB was similar to the control group. Seedlings submitted to mild salinity showed lower concentrations of ⋅OH and H_2_O_2_ when treated with GB or MT compared with the control group. Under mild and severe salinity treatments, the matrix table of the Tukey’s HSD test showed significant decrease in the H_2_O_2_ concentration by GB treatment in comparison with MT treatment. For 0.05 mM MT versus 50 mM GB under mild salinity, the P-value was equal to 0.008 (Table 2 and Supplementary Table 2). When the MT treatment was increased to 0.1 mM, the table showed a similar effect on plants treated with 50 mM GB. Seedlings under severe salt stress and treated with MT (M1 and M2) and 10 mM GB showed strong reduction in the ⋅OH content compared with seedlings subjected to 50 mM GB. Under mild salinity, the MDA levels were similar among all treatments. However, for seedlings subjected to 10 mM GB, the MDA content decreased strongly compared with the control. Although treatments with MT and GB at all levels during severe salinity reduced the MDA content in *D. odorifera* seedlings, the matrix table of the Tukey’s HDS concluded that GB treatments significantly decreased the MDA level in the seedlings compared with MT treatments under salinity (Table 2 and Supplementary Table 2).

**TABLE 2:** Two factor ANOVA (Salinity and GB/MT) for all parameters studied of *Dalbergia odorifera* significance values.

	Salinity factor	GB/MT factor	Interactions between salinity and GB/MT
Plant height increment	<0.0001****	<0.0001****	<0.0001****
Number of leaf increment	<0.0001****	<0.0001****	0.0236*
Stem of diameter increment	0.0455*	<0.0001****	0.0716 ns
Water content	<0.0001****	<0.0001****	0.0012**
Water potential	<0.0001****	<0.0001****	<0.0001****
Electrolyte leakage	<0.0001****	<0.0001****	<0.0001****
Leaf area	<0.0001****	<0.0001****	<0.0001****
Chlorophyll a	<0.0001****	<0.0001****	<0.0001****
Chlorophyll b	<0.0001****	<0.0001****	<0.0001****
Carotenoid	<0.0001****	<0.0001****	<0.0001****
Total Chlorophyll	<0.0001****	<0.0001****	<0.0001****
Pn	<0.0001****	<0.0001****	<0.0001****
Trr	<0.0001****	<0.0001****	<0.0001****
Gs	<0.0001****	<0.0001****	<0.0001****
Wue	<0.0001****	<0.0001****	<0.0001****
Ci	<0.0001****	<0.0001****	<0.0001****
Proline	<0.0001****	<0.0001****	<0.0001****
Soluble sugar	<0.0001****	<0.0001****	<0.0001****
Total of phenol	<0.0001****	<0.0001****	<0.0001****
Proteins	<0.0001****	<0.0001****	<0.0001****
Thiols	0.0011**	0.0106*	0.1133 ns
MDA	<0.0001****	<0.0001****	<0.0001****
H_2_O_2_	<0.0001****	<0.0001****	<0.0001****
⋅OH	<0.0001****	<0.0001****	<0.0001****
AOX	<0.0001****	<0.0001****	<0.0001****
POD	<0.0001****	<0.0001****	<0.0001****
CAT	<0.0001****	<0.0001****	<0.0001****
SOD	<0.0001****	<0.0001****	<0.0001****
APX	<0.0001****	0.0937 ns	0.0090**

****p-value < 0.0001, **p-value < 0.01, *p-value < 0.05, ns *P*-value > 0.05, no significant.

### Antioxidant Activities

**FIGURE 6:**
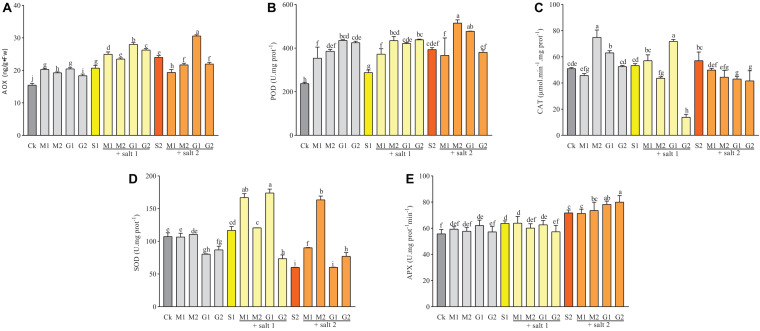
The activities of AOX **(A)**, POD **(B)**, SOD **(C)**, CAT **(D)**, and APX **(E)** in *D. odorifera* seedlings subjected to MT and GB under salt stress (150 and 250 mM). The bars on the top show SD, and different letters indicate significant differences according to Tukey’s multiple comparison test (*p* < 0.05).

Glycine betaine and MT significantly increased the AOX and POD activities in *D. odorifera* seedlings under well-watered and mild salinity conditions compared with the control. However, in severe salinity, only plants subjected to 10 mM GB exhibited an increase in AOX activity compared with the control group (Figure 6). The POD activities in salt stressed plants treated with 10 mM GB or 0.1 mM MT were significantly elevated compared with those in the control group S2 and the other groups, where the POD activity was similar. Based on the matrix table of the Tukey’s HDS (Supplementary Table 3), the AOX activity in plants subjected to 10 mM GB was better than that in plants treated with 0.05 mM MT under mild salinity. Furthermore, the 0.05 mM MT enhanced the AOX activity compared with 50 mM GB.

Mild salinity-treated plants showed a significant increase in the CAT and SOD activities when the seedlings were subjected to 0.05 mM MT and 10 mM GB compared with the control group (Figure 6). In severe salinity, only the SOD activity was enhanced by MT (M1 and M2) and GB at 50 mM. The seedlings of *D. odorifera* exhibited increased CAT activity under well-watered conditions and treatment with GB (G1 and G2) and MT at 0.1 mM compared with the control group. The SOD activity in seedlings treated with MT was similar to that in the control group but decreased significantly in plants treated with GB.

As shown in Supplementary Table 3, seedlings treated with MT increased the SOD activity under salt stress (*p*-value lower than 0.0001). However, seedlings treated with GB showed higher CAT activity compared with seedlings subjected to MT under mild salt stress (*p*-value equal to 0.0002). Figure 6 shows an increase in the APX activity in the leaves of salt-treated seedlings compared with that in well-watered plants. However, under salinity, only 50 mM GB-treated seedlings showed an increase in the APX activity compared with S2.

### Principal Component Analysis

**FIGURE 7:**
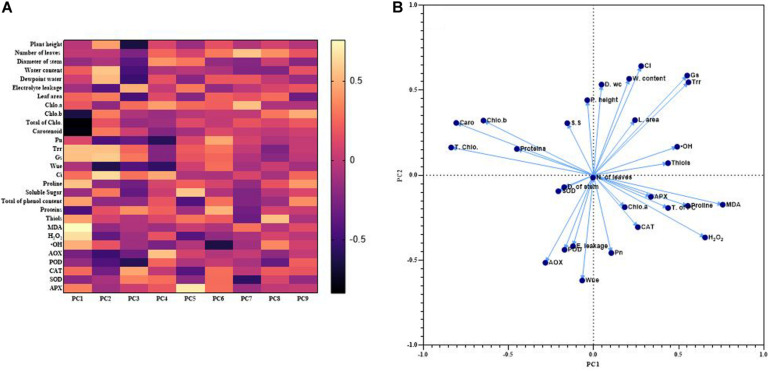
Heat map of correlations among various measured parameters based on the loadings PCAs **(A)** and plot of the first two PCAs showing association between various measured parameters **(B)**.

The first nine PCAs from loadings are used to draw a heat map that showed the correlation between different measured parameters. The total cumulative proportion of variance of these nine factors corresponded to 78.09% and their eigenvalues were higher than 1. The proportion of variance of the first two principal components (PC1 and PC2) was 17.76 and 13.63% with eigenvalues equal to 5.15 and 3.95, respectively. Indeed, the most significant contribution to explain the correlation between the measured parameters comes from PC1 and PC2. Thus, each data from different parameters was plot with PC1 and PC2. The Figure 7 showed a positive association between Leaf area, Gs and Trr; among the photosynthetic pigments excepted Chlorophyll a; between MDA, H_2_O_2_, proline, APX, and phenols content; amongst AOX, POD and EL; between relative water content and Ci as indicated by the small obtuse angle between their vectors. Based on the heat map, PC2 showed a better perceptible view of the system. Indeed, it exhibited a positive correlation between the plant height, relative water content, dew-point water potential, total of chlorophyll, Trr, Gs and leaf area. Meanwhile the correlation was negative between the plant height and the antioxidants enzymes, MDA or the H_2_O_2_. The positive correlation between plant height and relative water content or dew point water potential was significantly higher, 0.47 and 0.58, respectively (Figure 7).

## Discussion

Plants react to salinity over a variety of biochemical and molecular mechanisms, and the effects of salt on plant can be determined based on growth rate, metabolic status, photosynthetic parameters, and other indicators. Exogenous application of GB or MT confers strong salt stress tolerance to plants in different mechanisms. GB or MT can affect positively the plant growth in diverse crop plants, such as in soybean (Malekzadeh, 2015; Wei et al., 2015). In this present work, under salt stress or not, seedlings treated with 0.05 mM MT did not show increase or decrease in the plant growth parameters compared with mild salt-stressed plants or well-watered seedlings. In a recent work, Zhong et al. (2020) concluded a benefit effect of 0.15 mM MT on grape seedling growth. Seedlings treated with 0.1 mM MT showed better effect on the growth traits of *D. odorifera* seedlings compared with 0.05 mM MT-treated and well-watered plants. Indeed, the optimum concentration of MT that could promote plant growth differs according to the plants studied (Li et al., 2019). Salt stress has a negative effect on plant growth hormones, resulting in reduced growth to permit the plant to cope with stress. In this present study, GB showed better ability to increase plant growth than MT.

Exogenous application of MT and GB can protect and improve the activity of photosystem II. Under salt stress, MT and GB can enhance the biosynthesis of chlorophyll and slow the rate of its decomposition (Annunziata et al., 2019; Li et al., 2019). In this work, GB significantly increased the total of chlorophyll content compared with MT under salinity. One of the effects of salt stress on plants is to reduce water loss by closing the stomata. The variation in Gs manifested that under mild salinity, seedlings treated with MT and GB improved the reopening of stomata, thereby increasing the net photosynthesis rate.

Osmoprotectants, such as proline or soluble sugar, participate in regulating osmotic pressure in the cytoplasm and stabilizing proteins and membranes when plants face an abiotic stress. MT pre-treatment can increase the contents of proline and soluble protein under cold stress (Zhang et al., 2017) and can also limit cellular red-ox disruption by osmo-protection through the regulation of proline homeostasis (Antoniou et al., 2017). Based on recent studies, controversy exists with regard to how MT regulates soluble sugar content in plants. Pre-treatment of *Gossypium hirsutumm* L. seeds with 20 and 50 μM MT can increase the contents of proline, soluble proteins, and sugars (Chen et al., 2020). This present experiment showed a decrease in the proline and soluble sugar contents when the seedlings were subjected to GB and MT under severe salinity. Salinity is supposed to increase the concentrations of proline and soluble sugars to regulate homeostasis. The decrease in the concentrations of proline and soluble sugars is probably due to the adequate supply of GB and MT that are exogenously applied to plant cells. MT and GB can directly scavenge ROS and stabilize osmotic differences between the surroundings of the cell and the cytosol. Furthermore, salt stress affects negatively the concentration of proteins in plant cells. The results showed that GB increased the concentration of proteins better than MT in *D. odorifera* seedlings under saline conditions. Indeed one of the roles of GB is to safeguard proteins against different environmental stresses (Yancey, 1994). To face oxidative damages, the plant cell can use passive detoxification with different molecules, such as thiols, to scavenge the ROS. Zagorchev et al. (2013) reported that thiols are probably involved in plant response to almost all stress factors and are a key to plant stress tolerance. He also related that the leaves of tomato sprayed with 100 μM MT every 5 days showed protection of plant cells from ROS-induced damage by regulating 2-cysteine peroxiredoxin and biosynthesis of S-compounds. And in this present work, thiols were not affected by the supply of MT or GB. The phenolic compounds are other antioxidant molecules that can play a major role by scavenging the ROS. The antioxidant activity of phenolic composites allowed hyacinth bean plants to face oxidative stress caused by salinity. In *Hypericum pruinatum*, phenolic compounds show a significant physiological role in salinity (D’Souza and Devaraj, 2010; Caliskan et al., 2017). The results showed that the total of phenol content was significantly increased by MT and GB treatments under mild salinity.

Salt stress as abiotic stress can trigger an overproduction and accumulation of ROS, which are responsible for oxidative stress. Among ROS, ⋅OH and H_2_O_2_ are the protagonists of oxidative damages to proteins, nucleic acids, and lipid peroxidation in plants during stress. H_2_O_2_ is more stable than the other ROS, such as ⋅OH (1ns) (Sies, 1993), and is a suitable indicator for evaluating oxidative stress in plant physiology. Several studies have indicated that exogenous MT application enhances stress tolerance by decreasing the ROS and MDA contents in *Avena nuda* under salt stress (Gao et al., 2019), in *Pisum sativum* against oxidative stress (Szafrańska et al., 2016), and in wheat plant under cadmium stress (Ni et al., 2018). In this present work, MT and GB effectively reduced the concentrations of ROS, MDA, and the EL in fragrant rosewood leaves under saline conditions. A previous report revealed that seeds under salt stress treated with 20 mM MT showed a significant decrease in the MDA and EL levels in cotton seeds (Chen et al., 2020). Under severe salinity, both concentrations 0.05 mM and 0.1 mM of MT were suitable to decrease the MDA and EL contents in *D. odorifera* seedlings. Based on the results, GB treated-seedlings showed higher decrease in the levels of MDA, EL, and ROS compared with MT-treated seedlings under salinity. This phenomenon may be explained by two possibilities: either the concentrations of MT used are insufficient or excessive compared with the concentrations of GB used; or either GB is more efficient to decrease and scavenge ROS and MDA in fragrant rosewood seedlings under salt stress compared with MT.

**FIGURE 8:**
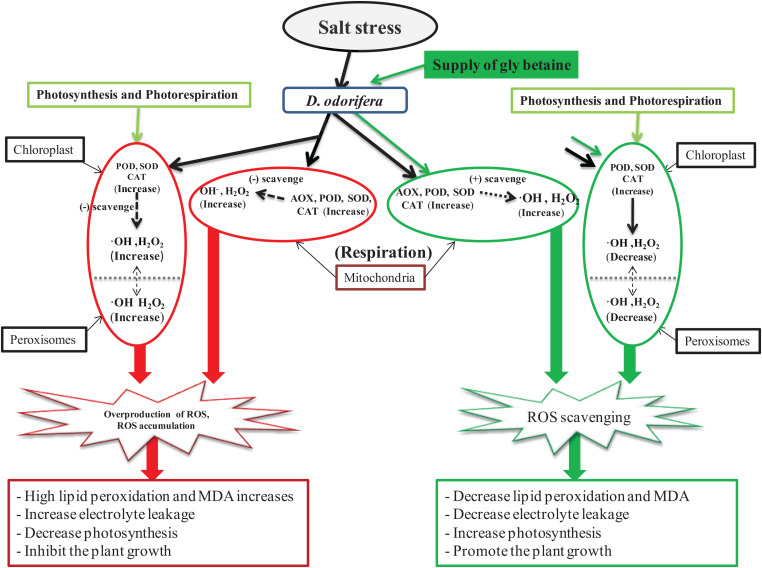
A model of diagram displaying the ability of GB scavenging the ROS and promoting salt stress tolerance in *D. odorifera* plants.

As one of the most threatening abiotic stresses in plants, salinity can cause perturbation in principal plant metabolic processes, such as photosynthesis, cellular respiration, and photorespiration, which stimulate the overproduction of ROS (Mittler et al., 2004). To counter the attack of the oxidative stress caused by overproduction and accumulation of ROS, plants can use enzymes, such as SOD, POD, CAT, APX, or AOX. These enzymes can scavenge ROS and protect the plant against oxidative damages. Several studies showed that exogenous application of MT or GB increases antioxidant enzyme activities under abiotic stress. In grape seedling leaves, exogenous MT treatment can significantly increase the CAT, SOD, and POD activities (Zhong et al., 2020). Exogenous application of GB can increase the activities of SOD, CAT, APX, or POD in wheat leaves against drought and salinity (Ma et al., 2006; Raza et al., 2007, 2014) as well as in *Glycine max* against salt stress (Malekzadeh, 2015). Furthermore, studies have proven that MT triggers the production of antioxidant enzymes in response to heat, salinity, and cold stress in *Lycopersicon esculemtum* (Martinez et al., 2018) and in tea (Li et al., 2018). In this present experiment, the results showed that exogenous application of GB and MT increased the POD, SOD, and SOD activities. However, the APX activity was constant and was not affected by the supply of exogenous MT or GB. Seedlings treated with 10 mM GB showed an increase in the POD, SOD, and CAT activities under mild salinity and increase in the POD activity under severe salinity. This finding suggests that a supply of GB can better improve antioxidant enzyme activities in *D. odorifera* seedlings compared with the other treatments under salt stress. One of the peculiarities of this study is the evaluation of AOX activity, which appears in few or none studies concerning the effect of MT or GB on salinity. The AOX can indirectly regulate homeostasis by directly reducing oxygen to water (Saha et al., 2016), and it is induced by different environmental stress factors (Vanlerberghe and McIntosh, 1997). The results of the present work showed that GB and MT increased the AOX activity under mild salinity, but only GB improved the AOX activity in fragrant rosewood seedlings under severe salinity. To summarize, we showed how GB and MT improved salt tolerance in fragrant rosewood seedlings. According to the data, GB promoted better salt tolerance in *D. odorifera* compared with MT. GB enhanced photosynthesis, improved redox homeostasis, decreased MDA accumulation, and increased the antioxidant enzyme activities. GB also contributed to scavenge ROS, thereby alleviating oxidative damages caused by salt stress in *D. odorifera* (Figure 8).

## Conclusion

Exogenous application of GB and MT efficiently relieved salinity damages in *D. odorifera* seedlings by scavenging ⋅OH, H_2_O_2_, and MDA. GB and MT promoted the growth parameters by enhancing photosynthetic and antioxidant activities and improved the salt stress tolerance by regulating antioxidant molecules, such as phenolic compounds, or osmolytes, such as proline and soluble sugar. The results confirmed the implication of exogenous MT and GB in improving AOX activity under salt stress. GB improved salt stress tolerance in *D. odorifera* seedlings better than MT. The synthesis of GB in plants has long been a target for engineering stress resistance. Hence, introducing the GB pathway might become a possibility for *D. odorifera* under saline stress.

## Data Availability Statement

The original contributions presented in the study are included in the article/Supplementary Material, further inquiries can be directed to the corresponding author.

## Author Contributions

All authors were engaged in this present work. EC and FY designed the experiment. EC performed most of the experiment and wrote the draft of the manuscript. L-FM, D-DL, J-FH, and JZ assist to carry the experiment in the greenhouse. J-FH assisted to perform the antioxidant enzymes analysis. FY provided funding, edited and revised the manuscript.

## Conflict of Interest

The authors declare that the research was conducted in the absence of any commercial or financial relationships that could be construed as a potential conflict of interest.

## References

[B1] Acosta-Motos J. Ortuño M. Bernal-Vicente A. Diaz-Vivancos P. Sanchez-Blanco M. Hernandez J. (2017). Plant responses to salt stress: adaptive mechanisms. *Agronomy* 7:18 10.3390/agronomy7010018

[B2] Aebi H. (1984). Catalase in vitro. *Method. Enzymol.* 105, 121–126. 10.1016/S0076-6879(84)05016-3 6727660

[B3] Annunziata M. G. Ciarmiello L. F. Woodrow P. Dell’Aversana E. Carillo P. (2019). Spatial and temporal profile of glycine betaine accumulation in plants under abiotic stresses. *Front. Plant Sci.* 10:230. 10.3389/fpls.2019.00230 30899269 PMC6416205

[B4] Antoniou C. Chatzimichail G. Xenofontos R. Pavlou J. J. Panagiotou E. Christou A. (2017). Melatonin systemically ameliorates drought stress-induced damage in Medicago sativa plants by modulating nitro-oxidative homeostasis and proline metabolism. *J. Pineal Res.* 62 12401. 10.1111/jpi.12401 28226194

[B5] Arnao M. B. Hernández-Ruiz J. (2019). Melatonin: a new plant hormone and/or a plant master regulator? *Trends Plant Sci.* 24 38–48. 10.1016/j.tplants.2018.10.010 30446305

[B6] Bates L. S. Waldren R. P. Teare I. D. (1973). Rapid determination of free proline for water-stress studies. *Plant Soil* 39, 205–207. 10.1007/BF00018060

[B7] Caliskan O. Radusiene J. Temizel K. E. Staunis Z. Cirak C. Kurt D. (2017). The effects of salt and drought stress on phenolic accumulation in greenhouse-grown Hypericum pruinatum. *Ital. J. Agron.* 12 271–275. 10.4081/ija.2017.918

[B8] Chen L. Liu L. Lu B. Ma T. Jiang D. Li J. (2020). Exogenous melatonin promotes seed germination and osmotic regulation under salt stress in cotton (*Gossypium hirsutum* L.). *PLoS One* 15:e0228241 10.1371/journal.pone.0228241 PMC6994006 32004326

[B9] Demiral T. Türkan I. (2004). Does exogenous glycinebetaine affect antioxidative system of rice seedlings under NaCl treatment? *J. Plant Physiol.* 161 1089–1100. 10.1016/j.jplph.2004.03.009 15535118

[B10] D’Souza M. R. Devaraj V. R. (2010). Biochemical responses of Hyacinth bean (*Lablab purpureus*) to salinity stress. *Acta Physiol. Plant.* 32 341–353. 10.1007/s11738-009-0412-2

[B11] Ellman G. L. (1959). Tissue sulfhydryl groups. *Arch. Biochem. Biophys.* 82, 70–77. 10.1016/0003-9861(59)90090-6 13650640

[B12] Fielding J. L. Hall J. L. (1978). A biochemical and cytochemical study of peroxidase activity in roots of *Pisum sativum*. *J. Exp. Bot.* 29, 983–991. 10.1093/jxb/29.4.983 12432039

[B13] Flowers T. S. Yeo A. R. (1989). “Effects of salinity on plant growth and crop yields,” in *Environmental Stress in Plants*, ed. Cherry J. H. (Berlin: Springer), 101–119. 10.1007/978-3-642-73163-1_11

[B14] Gao W. Feng Z. Bai Q. He J. Wang Y. (2019). Melatonin-mediated regulation of growth and antioxidant capacity in salt-tolerant naked oat under salt stress. *Int. J. Mol. Sci.* 20 1176. 10.3390/ijms20051176 30866540 PMC6429221

[B15] Hernández J. A. (2019). Salinity tolerance in plants: trends and perspectives. *Int. J. Mol. Sci.* 20 2408. 10.3390/ijms20102408 31096626 PMC6567217

[B16] Kolář J. Johnson C. H. Macháčková I. (2003). Exogenously applied melatonin (N -acetyl-5-methoxytryptamine) affects flowering of the short-day plant *Chenopodium rubrum*. *Physiol. Plant.* 118 605–612. 10.1034/j.1399-3054.2003.00114.x 11841302

[B17] Li J. Liu J. Zhu T. Zhao C. Li L. Chen M. (2019). The role of melatonin in salt Stress responses. *Int. J. Mol. Sci.* 20 1735. 10.3390/ijms20071735 30965607 PMC6479358

[B18] Li J. Zeng L. Cheng Y. Lu G. Fu G. Ma H. (2018). Exogenous melatonin alleviates damage from drought stress in *Brassica napus* L. (rapeseed) seedlings. *Acta Physiol. Plant.* 40:43 10.1007/s11738-017-2601-8

[B19] Li X. Wei J.-P. Scott E. Liu J.-W. Guo S. Li Y. (2018). Exogenous melatonin alleviates cold stress by promoting antioxidant defense and redox homeostasis in *Camellia sinensis* L. *Molecules* 23 165. 10.3390/molecules23010165 29342935 PMC6017414

[B20] Li X.-W. Chen Q.-X. Lei H.-Q. Wang J.-W. Yang S. Wei H.-X. (2018). Nutrient uptake and utilization by fragrant rosewood (*Dalbergia odorifera*) seedlings cultured with oligosaccharide addition under different lighting spectra. *Forests* 9:29 10.3390/f9010029

[B21] Lichtenthaler H. K. Wellburn A. R. (1983). Determinations of total carotenoids and chlorophylls a and b of leaf extracts in different solvents. *Biochem. Soc. T.* 11, 591–592. 10.1042/bst0110591

[B22] Liu F. Hong Z. Xu D. Jia H. Zhang N. Liu X. (2019). Genetic diversity of the endangered *Dalbergia odorifera* revealed by SSR markers. *Forests* 10:225 10.3390/f10030225

[B23] Ma Q.-Q. Wang W. Li Y.-H. Li D.-Q. Zou Q. (2006). Alleviation of photoinhibition in drought-stressed wheat (*Triticum aestivum*) by foliar-applied glycinebetaine. *J. Plant Physiol.* 163 165–175. 10.1016/j.jplph.2005.04.023 16399007

[B24] Malekzadeh P. (2015). Influence of exogenous application of glycinebetaine on antioxidative system and growth of salt-stressed soybean seedlings (*Glycine max* L.). *Physiol. Mol. Biol. Plants* 21 225–232. 10.1007/s12298-015-0292-4 25964715 PMC4411384

[B25] Martinez V. Nieves-Cordones M. Lopez-Delacalle M. Rodenas R. Mestre T. Garcia-Sanchez F. (2018). Tolerance to stress combination in tomato plants: new insights in the protective role of melatonin. *Molecules* 23 535. 10.3390/molecules23030535 29495548 PMC6017353

[B26] McNeil S. D. Nuccio M. L. Hanson A. D. (1999). Betaines and related osmoprotectants. Targets for metabolic engineering of stress resistance. *Plant Physiol.* 120 945–949. 10.1104/pp.120.4.945 10444077 PMC1539222

[B27] Mittler R. Vanderauwera S. Gollery M. Van Breusegem F. (2004). Reactive oxygen gene network of plants. *Trends Plant Sci.* 9 490–498. 10.1016/j.tplants.2004.08.009 15465684

[B28] Ni J. Wang Q. Shah F. Liu W. Wang D. Huang S. (2018). Exogenous melatonin confers cadmium tolerance by counterbalancing the hydrogen peroxide homeostasis in wheat seedlings. *Molecules* 23 799. 10.3390/molecules23040799 29601513 PMC6017192

[B29] Raza M. A. Saleem M. Shah G. Khan I. Raza A. (2014). Exogenous application of glycinebetaine and potassium for improving water relations and grain yield of wheat under drought. *J. Soil Sci. Plant Nutr.* 14 348–364. 10.4067/S0718-95162014005000028 27315006

[B30] Raza S. H. Athar H. R. Ashraf M. Hameed A. (2007). Glycinebetaine-induced modulation of antioxidant enzymes activities and ion accumulation in two wheat cultivars differing in salt tolerance. *Environ. Exp. Bot.* 60 368–376. 10.1016/j.envexpbot.2006.12.009

[B31] Saha B. Borovskii G. Panda S. K. (2016). Alternative oxidase and plant stress tolerance. *Plant Signal. Behav.* 11 e125630. 10.1080/15592324.2016.1256530 27830987 PMC5225930

[B32] Sies H. (1993). Strategies of antioxidant defense. *Eur. J. Biochem.* 215 213–219. 10.1111/j.1432-1033.1993.tb18025.x 7688300

[B33] Swain T. Hillis W. E. (1959). The phenolic constituents of *Prunus domestica. I.* — The quantitative analysis of phenolic constituents. *J. Sci. Food Agr.* 10, 63–68. 10.1002/jsfa.2740100110

[B34] Szafrańska K. Reiter R. J. Posmyk M. M. (2016). Melatonin application to pisum sativum L. seeds positively influences the function of the photosynthetic apparatus in growing seedlings during paraquat-induced oxidative stress. *Front. Plant Sci.* 7:1663. 10.3389/fpls.2016.01663 27867393 PMC5096385

[B35] Vanlerberghe G. C. McIntosh L. (1997). Alternativeoxidase: from gene to function. *Annu. Rev. Plant Physiol. Plant Mol. Biol.* 48 703–734. 10.1146/annurev.arplant.48.1.703 15012279

[B36] Volkmar K. M. Hu Y. Steppuhn H. (1998). Physiological responses of plants to salinity: a review. *Can. J. Plant Sci.* 78 19–27. 10.4141/P97-020

[B37] Wei W. Li Q.-T. Chu Y.-N. Reiter R. J. Yu X.-M. Zhu D.-H. (2015). Melatonin enhances plant growth and abiotic stress tolerance in soybean plants. *Journal of Experimental Botany* 66 695–707. 10.1093/jxb/eru392 25297548 PMC4321538

[B38] Yancey P. H. (1994). “Compatible and counteracting solutes,” in *Cellular and Molecular Physiology of Cell Volume Regulation*, ed. Strange K. (Boca Raton, FL: CRC Press), 81–109. 10.1201/9780367812140-7

[B39] Yang F. Han C. Li Z. Guo Y. Chan Z. (2015). Dissecting tissue- and species-specific responses of two Plantago species to waterlogging stress at physiological level. *Environ. Exp. Bot.* 109 177–185. 10.1016/j.envexpbot.2014.07.011

[B40] Yang F. Wang Y. Miao L.-F. (2010). Comparative physiological and proteomic responses to drought stress in two poplar species originating from different altitudes. *Physiol. Plant.* 139 388–400. 10.1111/j.1399-3054.2010.01375.x 20444190

[B41] Ye J. Wang S. Deng X. Yin L. Xiong B. Wang X. (2016). Melatonin increased maize (*Zea mays* L.) seedling drought tolerance by alleviating drought-induced photosynthetic inhibition and oxidative damage. *Acta Physiol. Plant.* 38:48 10.1007/s11738-015-2045-y

[B42] Yemm E. W. Willis A. J. (1954). The estimation of carbohydrates in plant extracts by anthrone. *Biochem. J.* 57, 508—514. 10.1042/bj0570508 13181867 PMC1269789

[B43] Zagorchev L. Seal C. Kranner I. Odjakova M. (2013). A central role for thiols in plant tolerance to abiotic stress. *Int. J. Mol. Sci.* 14 7405–7432. 10.3390/ijms14047405 23549272 PMC3645693

[B44] Zhang Y. P. Xu S. Yang S. J. Chen Y. Y. (2017). Melatonin alleviates cold-induced oxidative damage by regulation of ascorbate–glutathione and proline metabolism in melon seedlings (*Cucumis melo* L.). *J. Hortic. Sci. Biotechnol.* 92 313–324. 10.1080/14620316.2016.1266915

[B45] Zhong L. Lin L. Yang L. Liao M. Wang X. Wang J. (2020). Exogenous melatonin promotes growth and sucrose metabolism of grape seedlings. *PLoS One* 15:e0232033. 10.1371/journal.pone.0232033 32324780 PMC7179914

