# Supplementary material for: Gly Betaine Surpasses Melatonin to Improve Salt Tolerance in Dalbergia odorifera
Source: Front Plant Sci. 2021 Feb 9;12:588847. doi: 10.3389/fpls.2021.588847 (PMC7900558; doi:10.3389/fpls.2021.588847)

Supplementary Material

# Supplementary Figures legend

Figure 1 Effect of MT and GB on the growth of fragrant rosewood seedlings (7 months 25 days old).

Figure 2 Effect of MT and GB on the growth of fragrant rosewood seedlings under mild salinity (7 months 25 days old).

Figure 3 Effect of MT and GB on the growth of fragrant rosewood seedlings under high salinity (7 months 25 days old).

# Supplementary Tables

Table 1 Matrix of Tukey's HSD between MT and GB treatments under salinity; Pn , Trr , Gs , and Total of chlorophyll (n=4). **** P value < 0.0001, *** P value < 0.001, ** P value < 0.01, * P value < 0.05, ns P value > 0.05 No significant

| Parameters | Treatments | Salinity 150 mmol/l | | | Salinity 250 mmol/l | | |
| --- | --- | --- | --- | --- | --- | --- | --- |
|  |  | **Significant?** | **Level of significance** | **P Value** | **Significant?** | **Level of significance** | **P Value** |
| Pn | **M1 vs G1** | Yes | **** | <0.0001 | Yes | **** | <0.0001 |
|  | **M1 vs G2** | No | ns | 0.7541 | Yes | **** | <0.0001 |
|  | **M2 vs G1** | Yes | **** | <0.0001 | Yes | **** | <0.0001 |
|  | **M2 vs G2** | Yes | **** | <0.0001 | Yes | **** | <0.0001 |
| Trr | **M1 vs G1** | Yes | * | 0.013 | Yes | **** | <0.0001 |
|  | **M1 vs G2** | Yes | * | 0.0375 | Yes | * | 0.0311 |
|  | **M2 vs G1** | Yes | **** | <0.0001 | Yes | **** | <0.0001 |
|  | **M2 vs G2** | Yes | **** | <0.0001 | Yes | **** | <0.0001 |
| Gs | **M1 vs G1** | No | ns | 0.9774 | No | ns | 0.7763 |
|  | **M1 vs G2** | No | ns | 0.9984 | No | ns | 0.9774 |
|  | **M2 vs G1** | Yes | * | 0.0264 | Yes | ** | 0.0029 |
|  | **M2 vs G2** | Yes | * | 0.013 | No | ns | 0.1699 |
| Wue | **M1 vs G1** | Yes | **** | <0.0001 | Yes | **** | <0.0001 |
|  | **M1 vs G2** | No | ns | 0.9837 | Yes | **** | <0.0001 |
|  | **M2 vs G1** | Yes | **** | <0.0001 | No | ns | 0.1689 |
|  | **M2 vs G2** | Yes | **** | <0.0001 | Yes | **** | <0.0001 |
| T. of Chlo | **M1 vs G1** | Yes | **** | <0.0001 | Yes | * | 0.0173 |
|  | **M1 vs G2** | Yes | **** | <00001 | Yes | **** | <0.0001 |
|  | **M2 vs G1** | Yes | **** | <0.0001 | Yes | ** | 0.0068 |
|  | **M2 vs G2** | Yes | **** | <0.0001 | Yes | *** | 0.0002 |

Table 2 Matrix of Tukey's HSD between MT and GB treatments under salinity; Soluble Sugar, H_2_O_2,_•OH and MDA the data are expressed as means of four replicates (n = 4). **** P value < 0.0001, *** P value < 0.001, ** P value < 0.01, * P value < 0.05, ns P value > 0.05 No significant.

| Parameters | Treatments | Salinity 150 mmol/l | | | Salinity 250 mmol/l | | |
| --- | --- | --- | --- | --- | --- | --- | --- |
|  |  | **Significant?** | **Level of significance** | **P Value** | **Significant?** | **Level of significance** | **P Value** |
| Soluble Sugar | **M1 vs G1** | Yes | **** | <0.0001 | Yes | **** | <0.0001 |
|  | **M1 vs G2** | Yes | **** | <0.0001 | Yes | **** | <0.0001 |
|  | **M2 vs G1** | Yes | **** | <0.0001 | Yes | **** | <0.0001 |
|  | **M2 vs G2** | Yes | **** | <0.0001 | Yes | **** | <0.0001 |
| H_2_O_2_ | **M1 vs G1** | Yes | **** | <0.0001 | Yes | **** | <0.0001 |
|  | **M1 vs G2** | Yes | *** | 0.0008 | Yes | **** | <0.0001 |
|  | **M2 vs G1** | Yes | **** | <0.0001 | Yes | **** | <0.0001 |
|  | **M2 vs G2** | Yes | **** | <0.0001 | Yes | **** | <0.0001 |
| •OH | **M1 vs G1** | No | ns | 0.1935 | Yes | **** | <0.0001 |
|  | **M1 vs G2** | Yes | **** | <0.0001 | Yes | * | 0.0212 |
|  | **M2 vs G1** | No | ns | 0.2197 | Yes | **** | <0.0001 |
|  | **M2 vs G2** | No | ns | 0.7723 | No | ns | 0.8507 |
| MDA | **M1 vs G1** | Yes | **** | <0.0001 | Yes | **** | <0.0001 |
|  | **M1 vs G2** | Yes | *** | 0.0009 | Yes | **** | <0.0001 |
|  | **M2 vs G1** | Yes | **** | <0.0001 | No | ns | 0.9736 |
|  | **M2 vs G2** | Yes | ** | 0.0077 | Yes | **** | <0.0001 |

Table 3 Matrix of Tukey's HSD between MT and GB treatments under salinity; AOX, POD, CAT and SOD (n=4). **** P value < 0.0001, *** P value < 0.001, ** P value < 0.01, * P value < 0.05, ns P value > 0.05 No significant

| Parameters | Treatments | Salinity 150 mmol/l | | | Salinity 250 mmol/l | | |
| --- | --- | --- | --- | --- | --- | --- | --- |
|  |  | **Significant?** | **Level of significance** | **P Value** | **Significant?** | **Level of significance** | **P Value** |
| AOX | **M1 vs G1** | Yes | **** | <0.0001 | Yes | **** | <0.0001 |
|  | **M1 vs G2** | Yes | * | 0.038 | Yes | **** | <0.0001 |
|  | **M2 vs G1** | Yes | **** | <0.0001 | Yes | **** | <0.0001 |
|  | **M2 vs G2** | Yes | **** | <0.0001 | No | ns | 0.962 |
| POD | **M1 vs G1** | No | ns | 0.1759 | Yes | *** | 0.0002 |
|  | **M1 vs G2** | Yes | * | 0.0334 | No | ns | 0.9676 |
|  | **M2 vs G1** | No | ns | 0.9734 | No | ns | 0.4147 |
|  | **M2 vs G2** | No | ns | 0.9997 | Yes | **** | <0.0001 |
| CAT | **M1 vs G1** | Yes | *** | 0.0002 | No | ns | 0.1762 |
|  | **M1 vs G2** | Yes | **** | <0.0001 | No | ns | 0.0673 |
|  | **M2 vs G1** | Yes | **** | <0.0001 | No | ns | 0.989 |
|  | **M2 vs G2** | Yes | **** | <0.0001 | No | ns | 0.8737 |
| SOD | **M1 vs G1** | No | ns | 0.3217 | Yes | **** | <0.0001 |
|  | **M1 vs G2** | Yes | **** | <0.0001 | Yes | ** | 0.0085 |
|  | **M2 vs G1** | Yes | **** | <0.0001 | Yes | **** | <0.0001 |
|  | **M2 vs G2** | Yes | **** | <0.0001 | Yes | **** | <0.0001 |


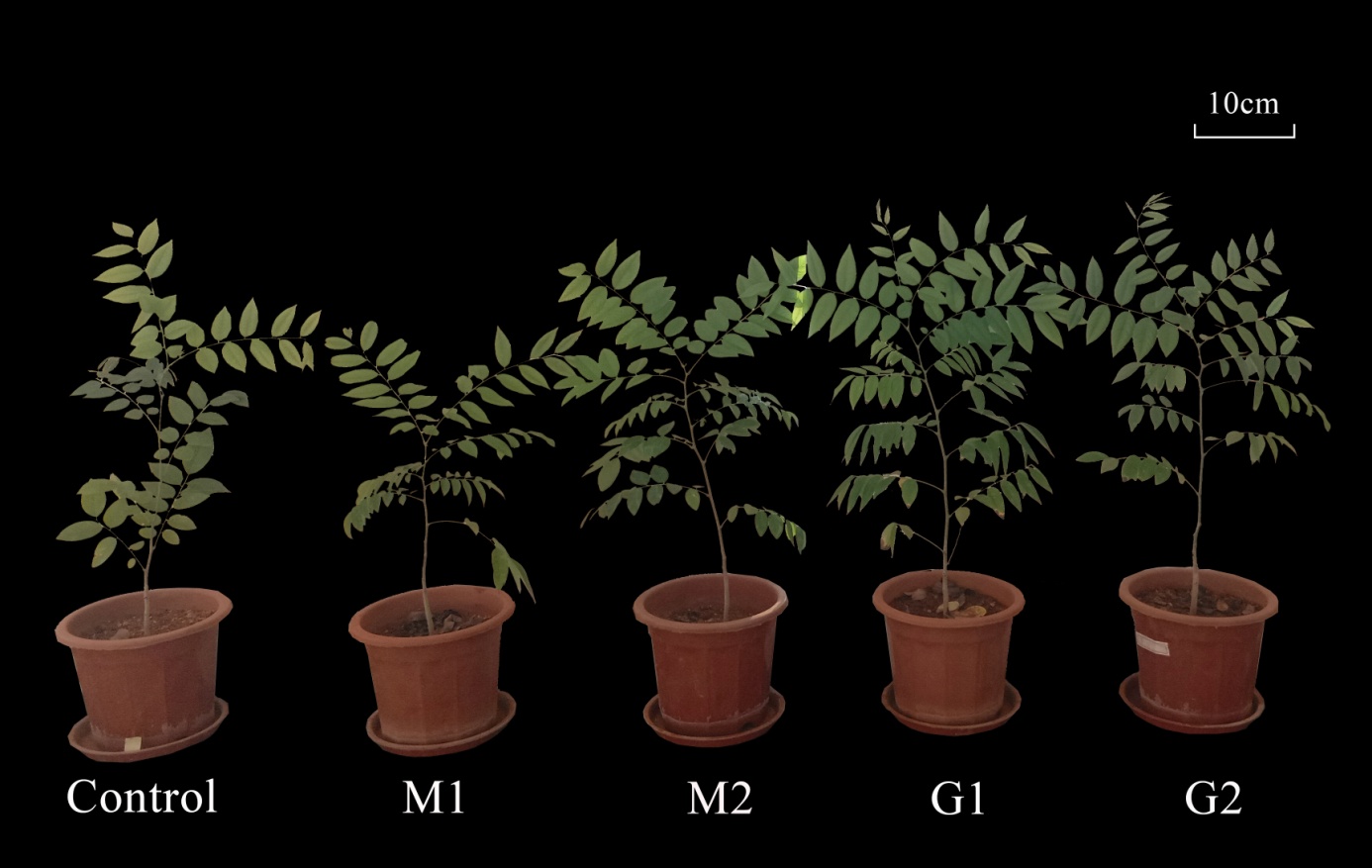


Fig. 1


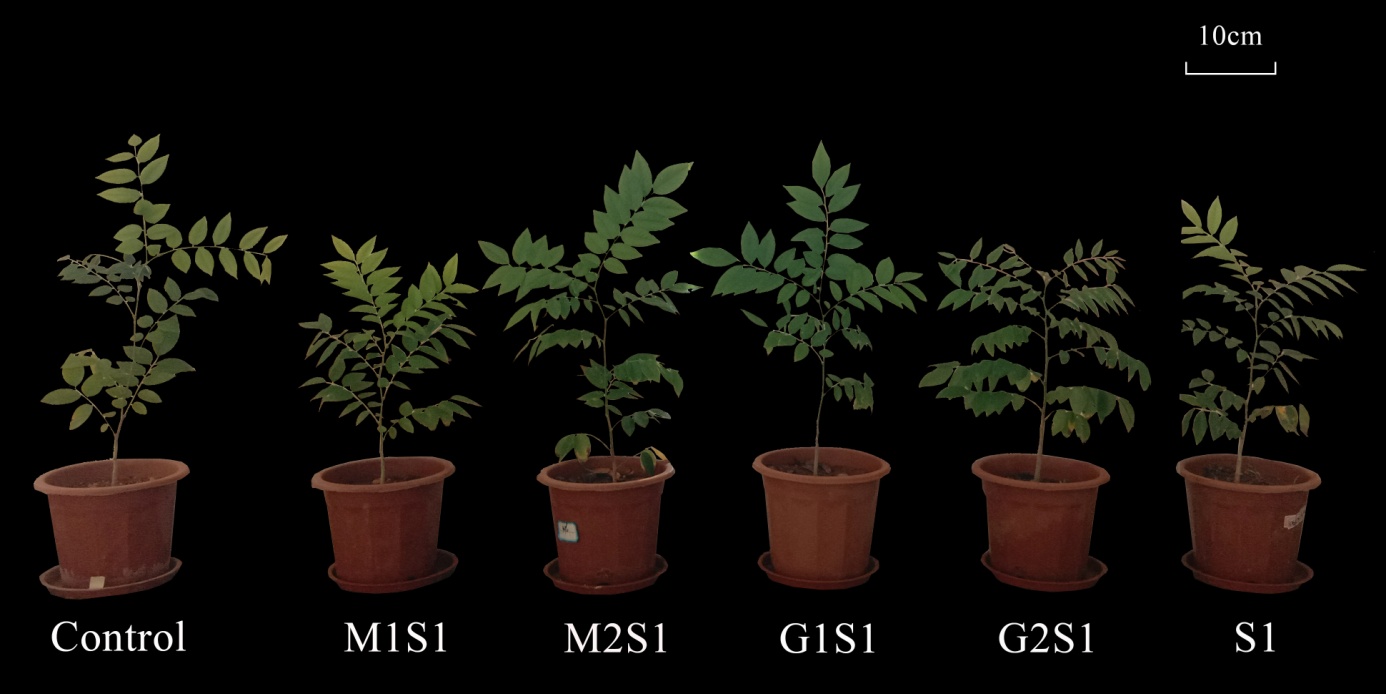


Fig. 2


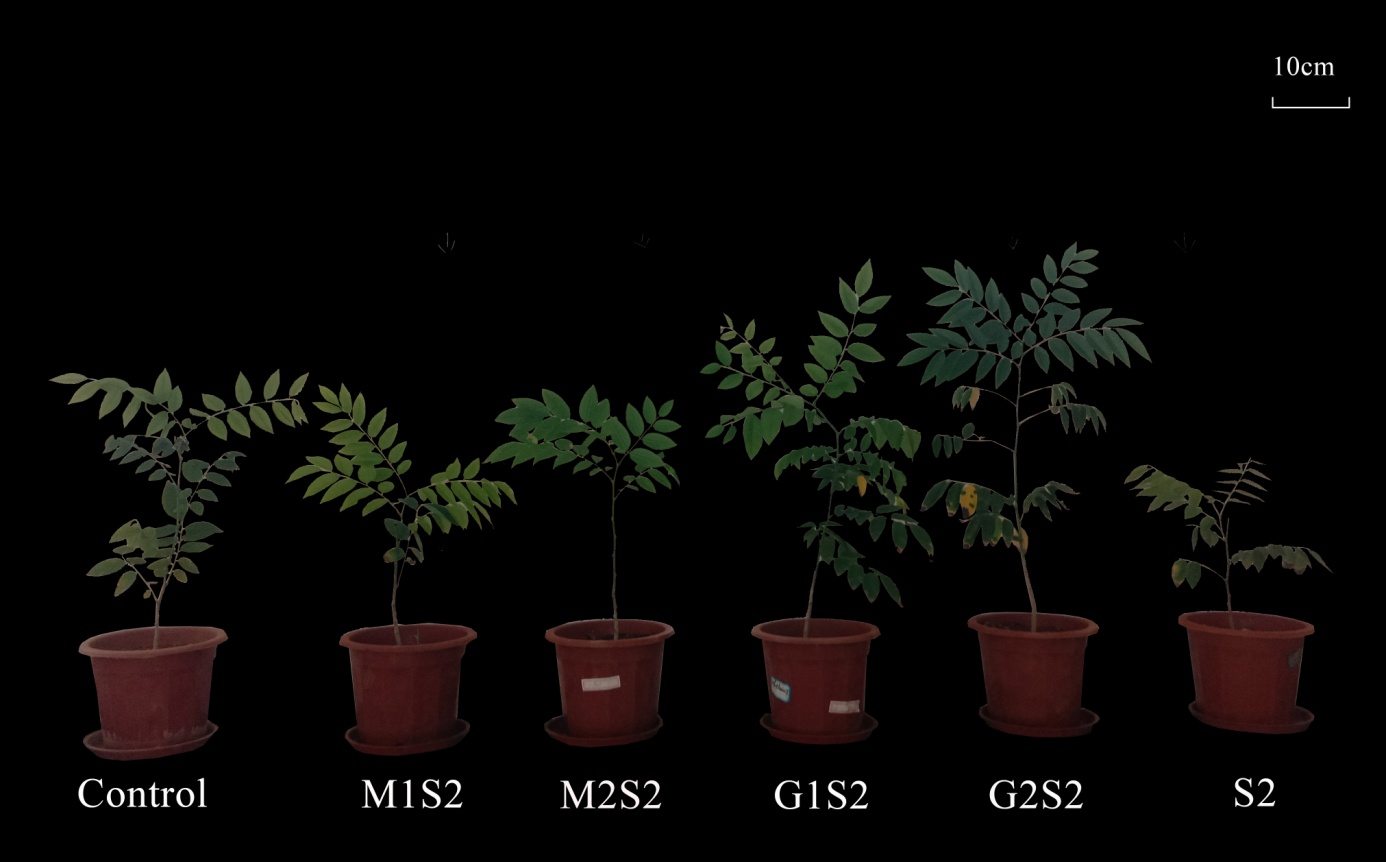


Fig. 3


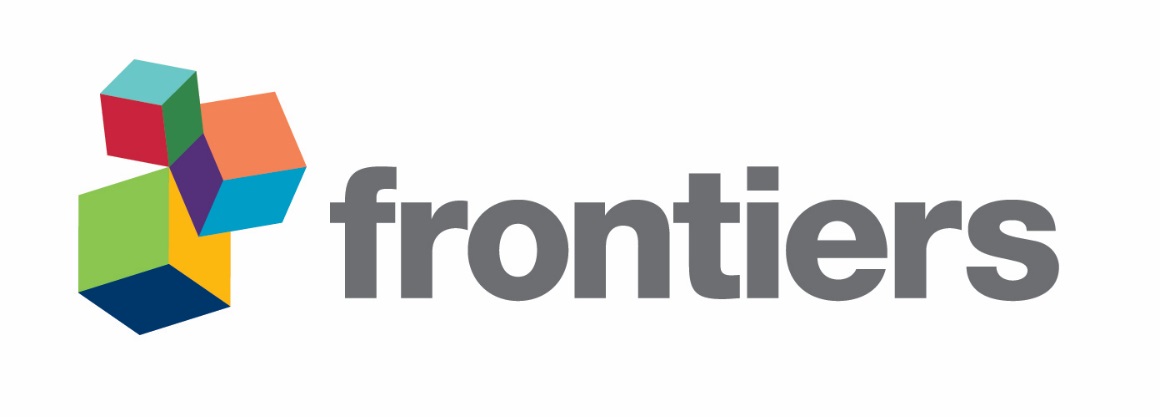

Supplement: Supplementary file 1 [file Data_Sheet_1.docx]
